# Supplementary figures and images for: Integrated analysis of microbiome and metabolome reveals signatures in PDAC tumorigenesis and prognosis
Source: Microbiol Spectr. 2024 Oct 10;12(11):e00962-24. doi: 10.1128/spectrum.00962-24 (PMC11540152; doi:10.1128/spectrum.00962-24)

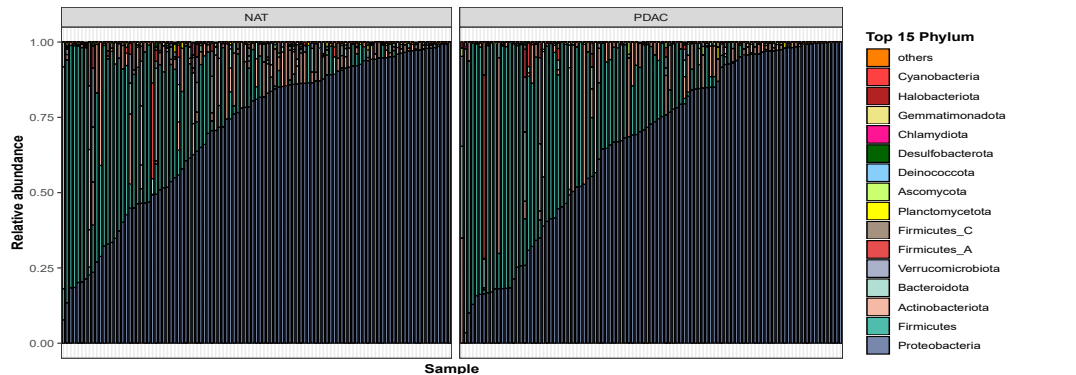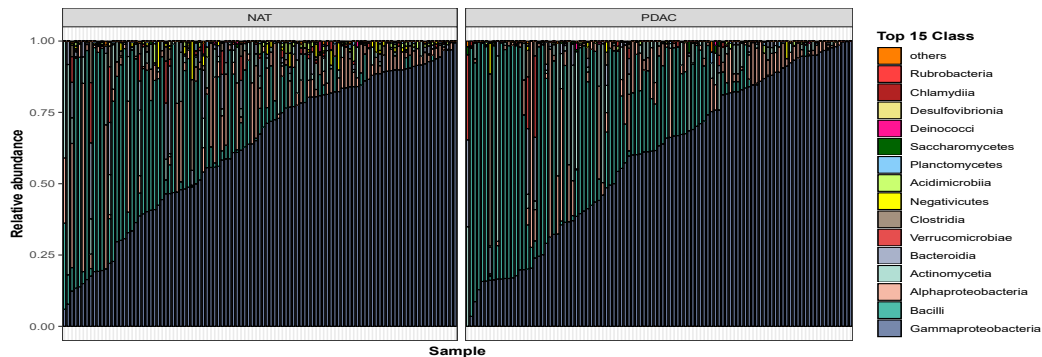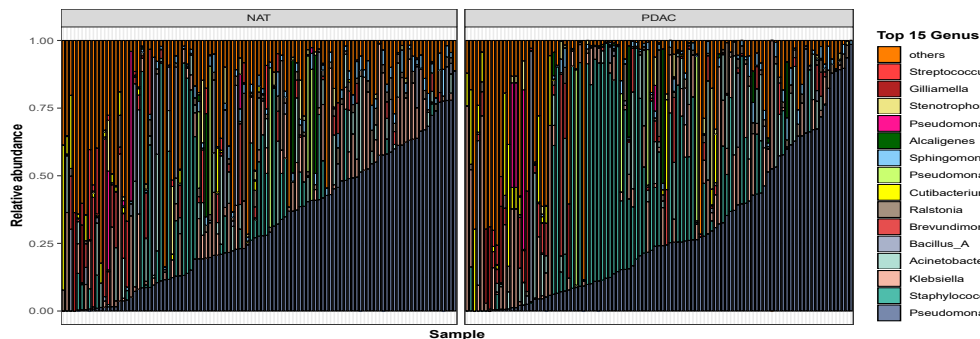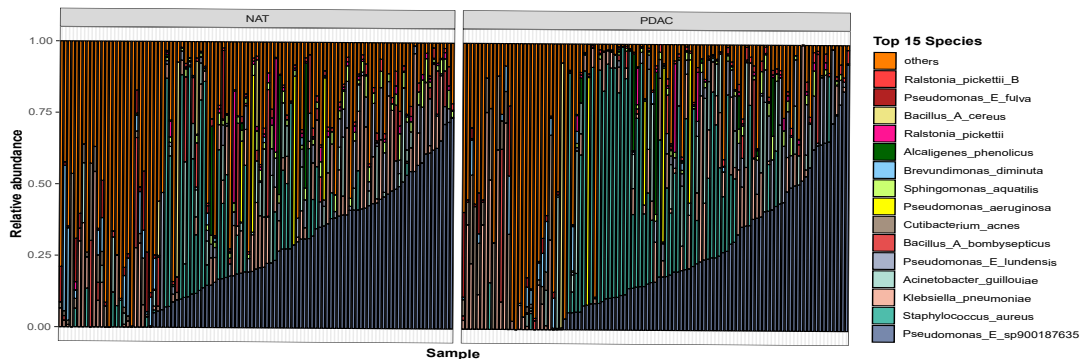

Supplement: Figure S1 — Stackplot of bacterial composition. [file spectrum.00962-24-s0002.pdf]

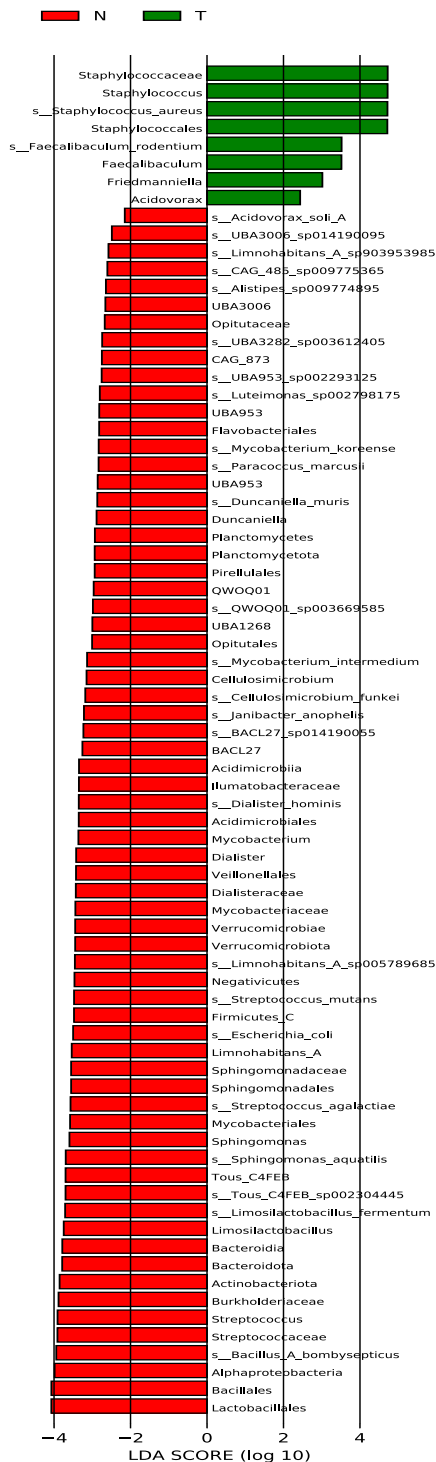

Supplement: Figure S2 — Microbial biomarkers identified by LEfSe analysis. [file spectrum.00962-24-s0003.pdf]

A

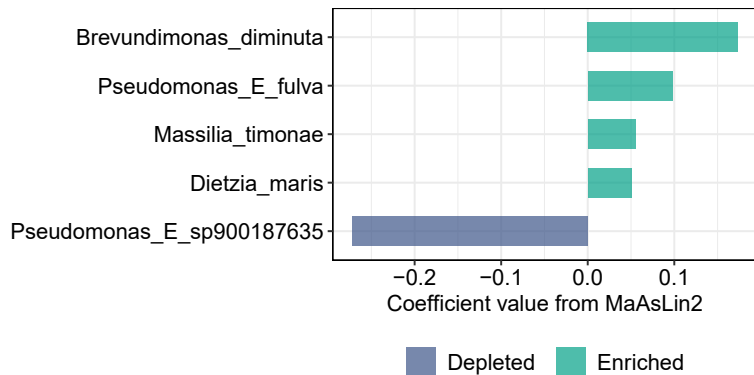

B

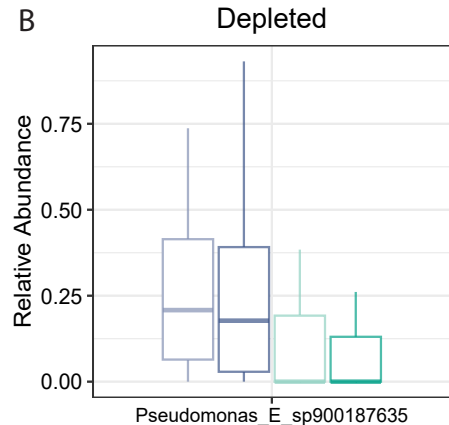

C

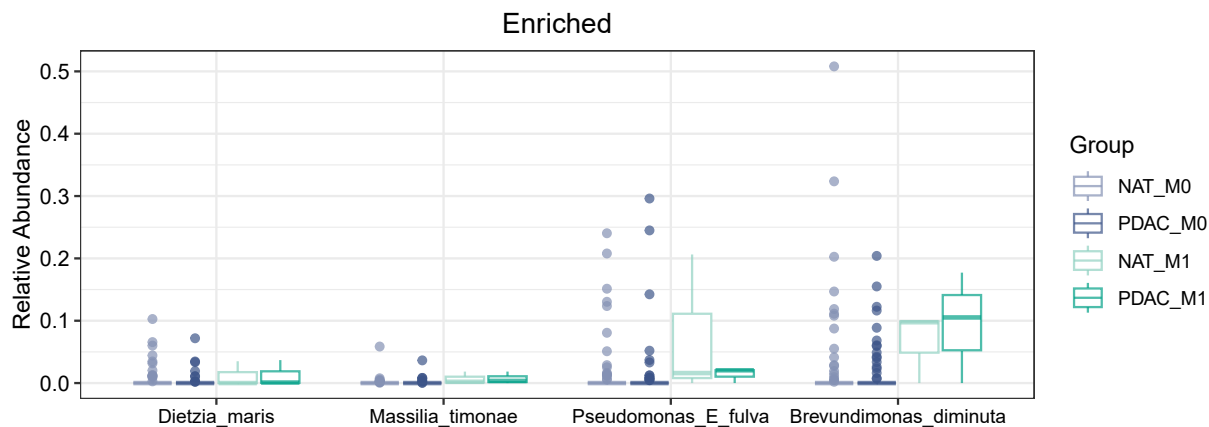

Supplement: Figure S3 — Differential bacterial species associated to tumor metastasis. [file spectrum.00962-24-s0004.pdf]

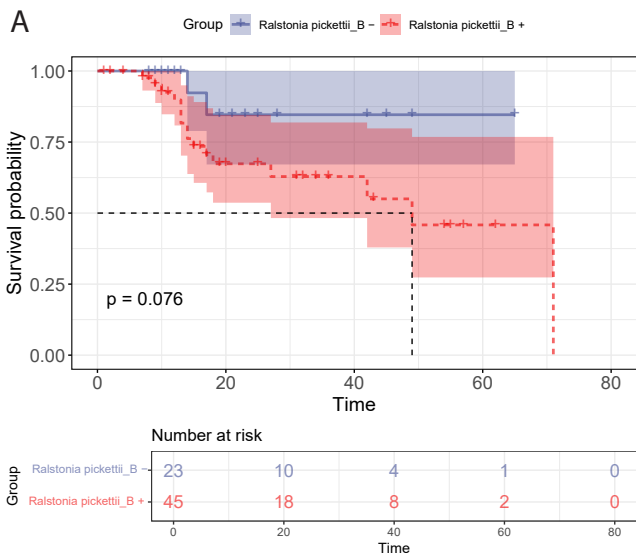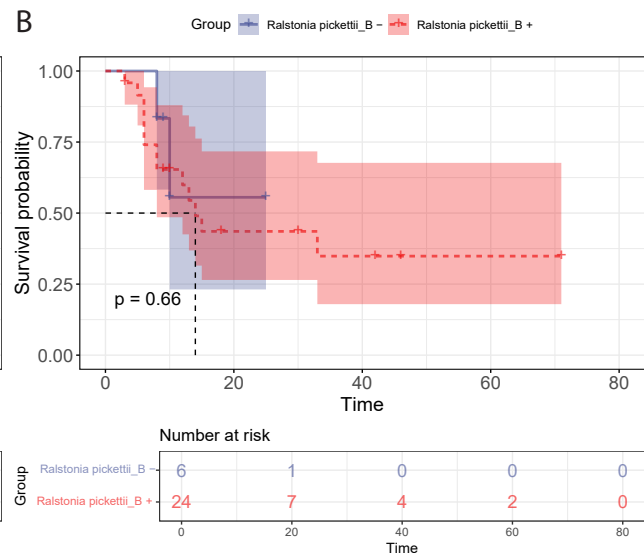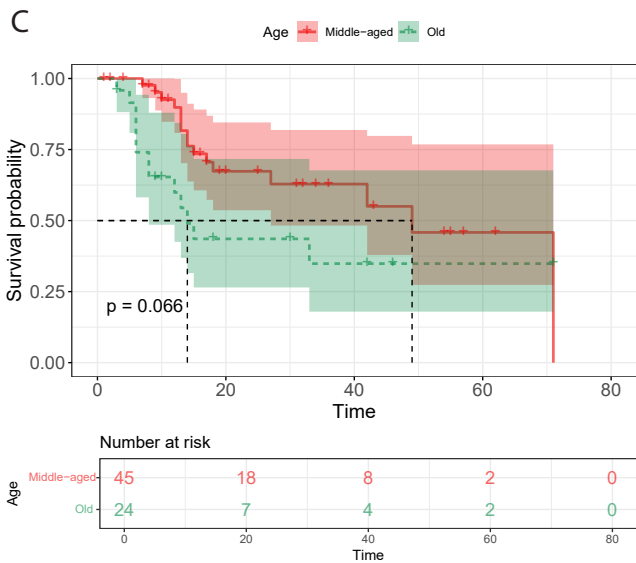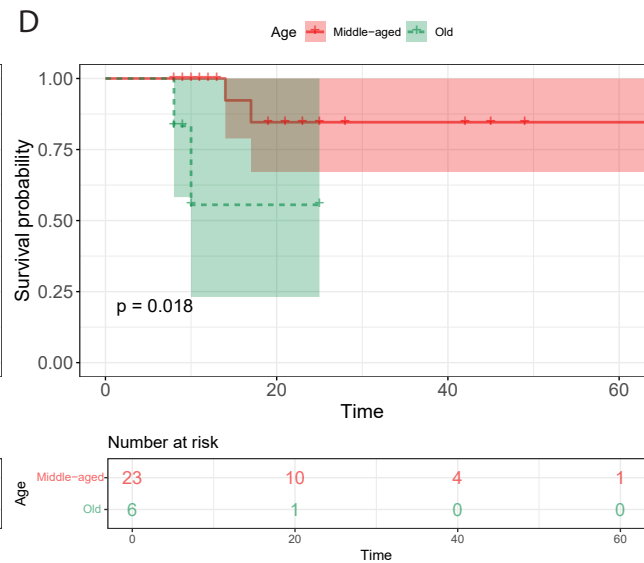

Supplement: Figure S4 — Subgroup survival Kaplan-Meier curve Presence of Ralstonia pickettii_B. [file spectrum.00962-24-s0005.pdf]

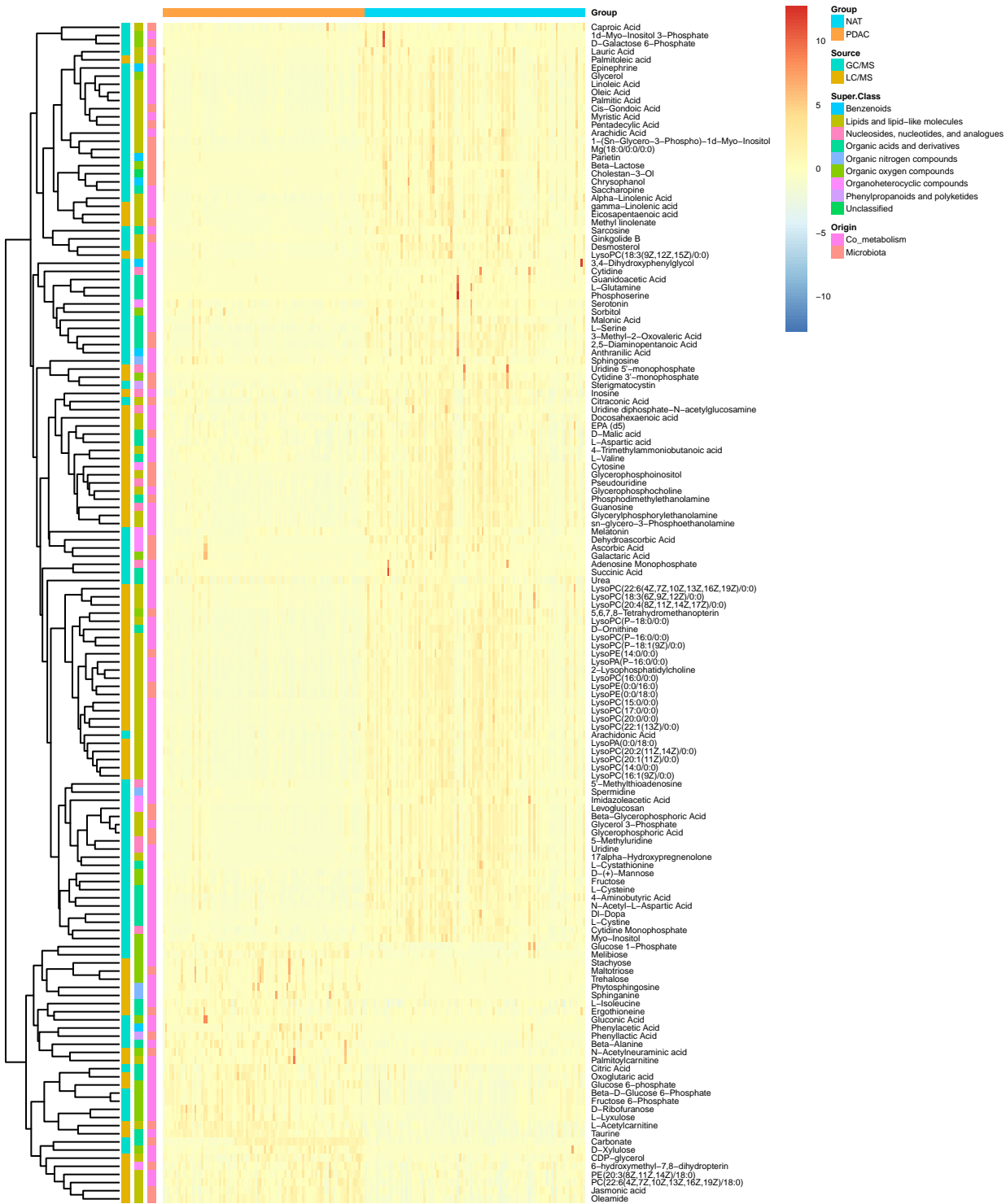

Supplement: Figure S5 — Heatmap of differential metabolites. [file spectrum.00962-24-s0006.pdf]

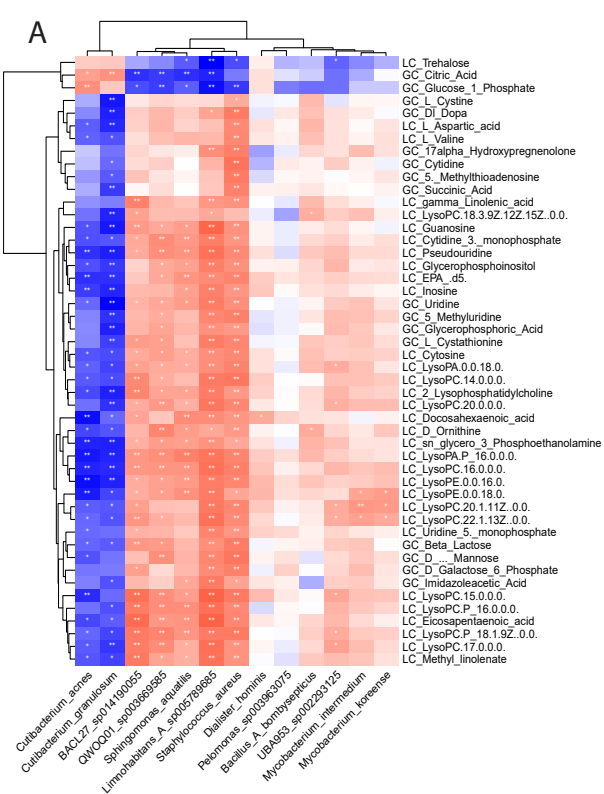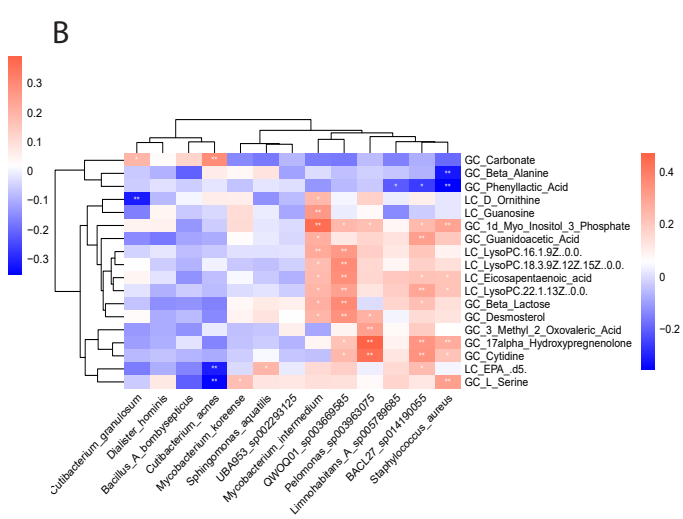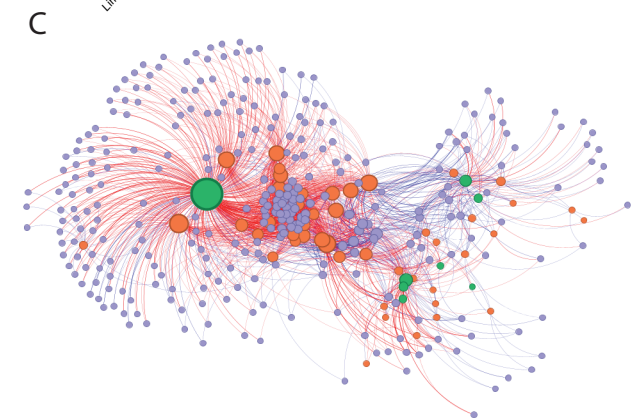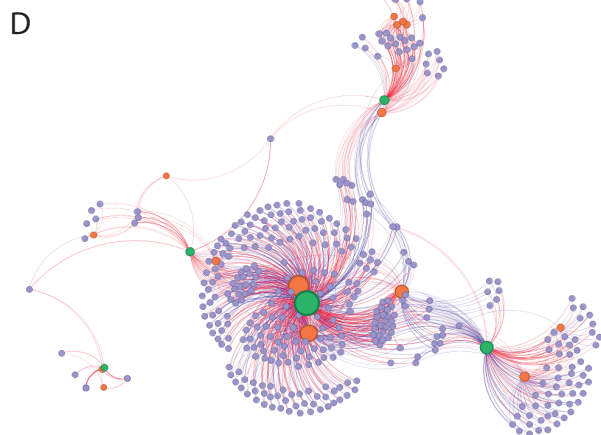

Supplement: Figure S6 — Correlation between differential metabolites. [file spectrum.00962-24-s0007.pdf]

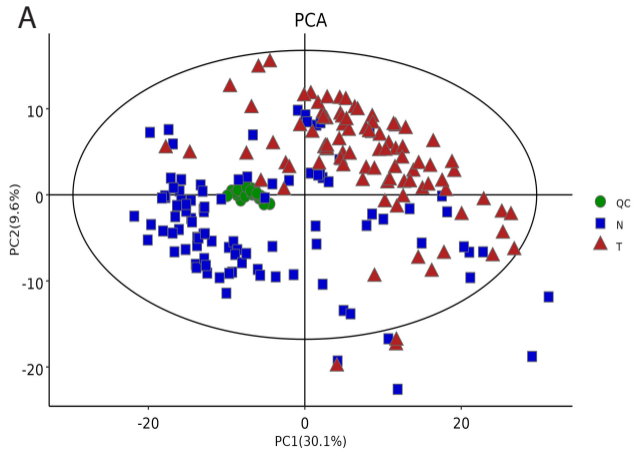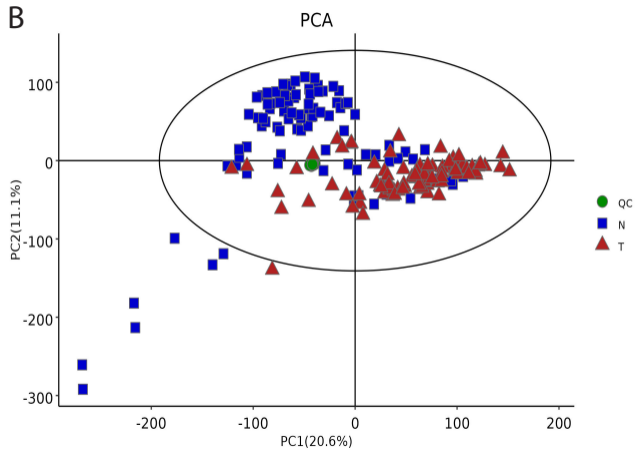

Supplement: Figure S7 — PCA plot of metabolome quality control. [file spectrum.00962-24-s0008.pdf]
