# Supplementary material for: Integrated analysis of microbiome and metabolome reveals signatures in PDAC tumorigenesis and prognosis
Source: Microbiol Spectr. 2024 Oct 10;12(11):e00962-24. doi: 10.1128/spectrum.00962-24 (PMC11540152; doi:10.1128/spectrum.00962-24)
Supplement: Supplemental material — Legends for supplemental figures and tables, and additional experimental details. [file spectrum.00962-24-s0009.docx]

Supplementary Materials for

Integrated Analysis of Microbiome and Metabolome Reveals Signatures in PDAC Tumorigenesis and Prognosis

Yuan Fang, Xiaohong Liu, Jie Ren, Xing Wang, Feihan Zhou, Shi Huang, Lei You^*^ and Yupei Zhao^*^.

Correspondence to: florayo@163.com; zhao8028@263.net

**This PDF file includes:**

Materials and Methods

Legend of Figures. S1 to S6

Table S1 to 3

Description of data S1

**Other Supplementary Materials for this manuscript include the following:**

Data. S1

Materials and Methods

DNA extraction, construction of amplicon library, and sequencing

Genomic DNA of PDAC tumors and NAT was extracted using a TIANamp Micro DNA Kit (Tiangen, cat. #DP316). DNA (1 pg to 200 ng) was digested 4 U of enzyme BcgI (NEB) for a duration of 3 h at 37℃. Following digestion, DNA fragments were ligated with adaptors. The ligation reaction involved combining 5 µL of digested DNA with 10 µL of a ligation master mix , which contained 0.2 µM of each adaptor and 800 U of T4 DNA ligase (New England Biolabs, NEB). The ligation was carried out at 4°C for 12 hours. Subsequently, the ligation products were amplified and the resulting PCR products were subjected to 8% polyacrylamide gel. Bands of approximately 100 bp were precisely excised from the polyacrylamide gel, and the DNA was diffused from the gel in nuclease-free water at 4℃ for 12 h. To introduce sample-specific barcodes, PCR was performed using platform-specific primers that contained barcodes. Each 20 µL PCR contained 25 ng of gel-extracted PCR product, 0.2 µM of each primer, 0.3 mM dNTP, 1×Phusion HF buffer and 0.4 U Phusion high-fidelity DNA polymerase (NEB). Following amplification, the PCR products were purified using a QIAquick PCR purification kit (Qiagen) and subsequently sequenced on the Illumina Nova PE150 platform. The 2bRAD-M sequencing was conducted at Qingdao OE Biotech Co., Ltd. (Qingdao, China).

Sequencing processing and decontamination

During the sequence quality control process, reads exhibiting an N base proportion greater than 8% and low-quality reads—defined as those having a base quality value below Q30, were excluded from further analysis. To identify the microbial species within each sample, the sequenced 2bRAD tags underwent quality control and were subsequently mapped against the 2bRAD marker database using a built-in Perl script. This database contains unique 2bRAD tags associated with 26,163 microbial species. To ensure accurate species identification and minimize false positives, a G-score was calculated for each identified species within a sample:

G score species i = √(S_i_ × t_i_ )

(S: number of reads assigned to all 2bRAD markers belonging to species i in a sample. t: number of all 2bRAD markers of species i that have been sequenced within a sample).

The G-score is a harmonic mean of the read coverage of 2bRAD markers specific to a species and the total number of possible 2bRAD markers for that species. A threshold G score of 5 was established to minimize false-positive discoveries of microbial species. The relative abundance of a specific species was determined by calculating the ratio of the number of microbial individuals attributed to that species to the total number of individuals from known species detectable within a given sample^(1)^. The present study employed air samples from laboratory and surgical environments as negative controls, utilizing a combination of decontam, microDecon, and FEAST to effectively eliminate background microorganisms from the experimental samples.

Tissue sample preparation for LC-MS untargeted metabolome analysis

To prepare the sample for analysis, 30 mg of the tissue sample was accurately weighed and transferred into a 1.5 mL EP tube. Subsequently, 600 μL of a methanol-water solution (V:V=4:1) containing L-2-chlorophenylalanine at a concentration of 4 μg/mL was added to the tube. To facilitate homogenization, two small steel balls were added to the tube, which was then pre-cooled in a -40°C freezer for 2 min. After pre-cooling, the sample was ground in a grinder at a frequency of 60 Hz for 2 minutes. For the extraction step, the sample was subjected to ultrasound treatment in an ice water bath for 10 minutes, followed by further cooling at -40°C for 2 hours to enhance extraction efficiency. Subsequently, the tube was centrifuged at 13,000 rpm and 4°C for 10 min. After centrifugation, 150 μL of supernatant was carefully aspirated using a syringe. To remove any particulate matter, the supernatant was filtered using a 0.22 μm organic phase pinhole filter. The resulting filtrate was transferred to LC injection vials and stored at -80°C until LC-MS analysis was performed ensure the reliability and reproducibility of the analysis. Quality Control Samples (QCs) were prepared by combining equal volumes of extract from all samples. These QCs served as reference standards for quality assessment during the analysis.

Tissue sample preparation for GC-MS untargeted metabolome analysis

To prepare the sample, 30 mg of the sample was weighed and placed in a 1.5 mL centrifuge tube along with two small steel balls. Next, 600 μL of a methanol-water solution (V:V=4:1) containing L-2-chlorophenylalanine at a concentration of 4 μg/mL was added. The tube was placed in a -40°C freezer for 2 min and subsequently ground in a grinder at a frequency of 60 Hz for 2 min. Next, 120 μL chloroform was added to the tube, followed by vortexing for 2 min. The sample was then subjected to ultrasound extraction in an ice water bath for 10 min and further cooled at -40°C for 30 min. After cooling, the tube was centrifuged at low temperature (13,000 rpm, 4°C) for 10 min. The supernatant (150 μL) was collected and transferred to a glass bottle. The sample was then concentrated using a centrifugal concentrator. Subsequently, 80 μL of methoxyamine hydrochloride pyridine solution (15 mg/mL) was added to the vial, followed by vortex mixing for 2 minutes. The vial was then incubated at 37°C in a shaking incubator for 60 minutes to facilitate the oxime reaction. Afterward, 50 μL of BSTFA derivatization reagent and 20 μL of n-hexane were added to the vial. Additionally, 10 internal standards (C8/C9/C10/C12/C14/C16/C18/C20/C22/C24, all in chloroform) were added in a volume of 10 μL. The reaction mixture was vortexed for 2 min and then incubated at 70°C for 60 min. After removing the samples, they were left at room temperature for 30 min prior to the GC-MS metabolomics analysis.

LC-MS/MS condition for untargeted metabolome analysis

The chromatographic conditions were as follows: the column used was ACQUITY UPLC HSS T3 (100 mm × 2.1 mm, 1.8 μm) and it was maintained at a temperature of 45°C. The mobile phase consisted of two components A, which was water containing 0.1% formic acid) and B, which was acetonitrile containing 0.1% formic acid). The flow rate was set at 0.35 mL/min, and the injection volume was 2 μL. ESI was used as the ion source for MS, and both positive and negative ion scanning modes were employed for sample signal acquisition.

GC-MS/MS condition for untargeted metabolome analysis

The experiment utilized a DB-5MS capillary column (30 m × 0.25 mm × 0.25 μm, Agilent J&W Scientific, Folsom, CA, USA) for separation. The carrier gas used was High-purity helium, with a minimum purity of 99.999%. The flow rate was set at 1.0 mL/min, and the inlet temperature was maintained at 260°C. A 1 μL injection volume was used without splitting, and a solvent delay of 5 min was implemented. The programmed heating protocol involved initially setting the column oven temperature at 60°C for 0.5 min, followed by an 8°C/min increase to 125°C, another 8°C/min increase to 210°C, a 15°C/min increase to 270°C, and finally a 20°C/min increase to 305°C, which was held for 5 min. An electron bombardment ion source (EI) was employed with a source temperature of 230°C and a quadrupole temperature of 150°C. The electron energy is 70 eV. The scanning mode utilized was the full scan mode (SCAN), covering a mass-to-charge ratio (m/z) range of 50-500.

Metabolome data processing

The original LC-MS data were processed using Progenesis QI V2.3 (Nonlinear, Dynamics, Newcastle, UK) for baseline filtering, peak identification, integration, retention time correction, peak alignment, and normalization. main parameters of 5 ppm precursor tolerance, 10 ppm product tolerance, and 5% product ion threshold were applied. Compound identification was based on the precise mass-to-charge ratio (M/z), secondary fragments, and isotopic distribution using The Human Metabolome Database (HMDB), Lipidmaps (V2.3), Metlin, EMDB, PMDB, and self-built databases for qualitative analysis. The extracted data were then further processed by removing any peaks with a missing value (ion intensity = 0) in more than 50% of groups, by replacing zero value by half of the minimum value, and by screening according to the qualitative results of the compound. Compounds with scores below 36 (out of 60) were also deemed inaccurate and removed. A data matrix was created from the positive and negative ion data.

The GC/MS raw data were obtained from. The D format is transferred to. abf format using the software Analysis Base File Converter for the quick retrieval of data. The data were then imported into MS-DIAL software, which performs peak detection, peak identification, MS2Dec deconvolution, characterization, peak alignment, wave filtering, and missing value interpolation. Metabolite characterization was based on the LUG database. A data matrix is derived. The three-dimensional matrix includes sample information, the name of the peak of each substance, retention time, retention index, mass-to-charge ratio, and signal intensity. For each sample, all peak signal intensities were segmented and normalized based on internal standards, with an RSD greater than 0.3 being retained after screening. Following normalization, redundancy was removed, and peak merging was conducted to create the final data matrix. This matrix was then imported into R for subsequent analysis.

1.Sun Z, Huang S, Zhu PF, Tzehau L, Zhao HL, Lv J, Zhang RC, Zhou LS, Niu QY, Wang XP, Zhang M, Jing GC, Bao ZM, Liu JQ, Wang S, Xu J. 2022. Species-resolved sequencing of low-biomass or degraded microbiomes using 2bRAD-M. Genome Biology 23.

Fig. S1. Stackplot of bacterial composition.(A) Phylum (B) Class (C) Genus (D) Species.

Fig. S2. Microbial biomarkers identified by LEfSe anlysis.

Fig. S3. Differential bacterial species associated to tumor metastasis. (A) Differential species associated to M stage identified by microbiome multivariable associations with linear model (MaAsLin 2) adjusted for confounding factors (*q < 0.25). (B, C) Boxplot showed relative abundance of bacterial species associated to M stage, (B) depleted, (C) enriched.

Fig. S4. Subgroup survival Kaplan-Meier curve Presence of *Ralstonia pickettii_B* in (A) middle-aged and (B) old group. Middle-aged and old in (C) *Ralstonia pickettii_B* positive and (D) negative group.

Fig. S5. Heatmap of differential metabolites originated from microbiota and host-microbiota co-metabolism.

Fig. S6. Correlation between differential metabolites, KO genes and bacterial species. Heatmap of significant correlation between differential metabolites and bacterial species in (A) NAT.(B) PDAC. *r > 0.3 or r <-0.3, and p < 0.05, ** r > 0.3 or r <-0.3, and p < 0.01. Networks of significant correlation of species-KO genes-metabolites in (C) NAT and (D )PDAC. Green nodes represent species, yellow for metabolites, and purple for KO genes. r > 0.3and p < 0.05.

Fig. S7. PCA plot of metabolome quality control. (A) GC-MS (B) LC-MS.

Table S1. Results of MaASLin2 on species level

| Species | value | coef | *P* | q-value |
| --- | --- | --- | --- | --- |
| *Sphingomonas aquatilis* | PDAC | -0.04723 | 7.97E-06 | 0.00051 |
| *Massilia timonae* | M1 | 0.054961 | 2.09E-05 | 0.000519 |
| *QWOQ01 sp003669585* | PDAC | -0.02142 | 2.43E-05 | 0.000519 |
| *BACL27 sp014190055* | PDAC | -0.02731 | 3.53E-05 | 0.000564 |
| *Dialister hominis* | PDAC | -0.02822 | 0.000153 | 0.001963 |
| *Limnohabitans_A sp005789685* | PDAC | -0.02959 | 0.000959 | 0.008814 |
| *Mycobacterium koreense* | PDAC | -0.01333 | 0.000964 | 0.008814 |
| *Brevundimonas diminuta* | M1 | 0.173556 | 0.001708 | 0.013662 |
| *Staphylococcus aureus* | PDAC | 0.148131 | 0.002429 | 0.017272 |
| *Mycobacterium intermedium* | PDAC | -0.01463 | 0.008544 | 0.054681 |
| *UBA953 sp002293125* | PDAC | -0.01004 | 0.02006 | 0.116712 |
| *Cutibacterium cnes* | PDAC | 0.027423 | 0.032739 | 0.161178 |
| *Pseudomonas_E fulva* | M1 | 0.097925 | 0.032479 | 0.161178 |
| *Bacillus_A bombysepticus* | PDAC | -0.04193 | 0.036937 | 0.166117 |
| *Dietzia maris* | M1 | 0.050594 | 0.038934 | 0.166117 |
| *Cutibacterium granulosum* | PDAC | 0.006172 | 0.051225 | 0.192848 |
| *Pelomonas sp003963075* | PDAC | -0.00575 | 0.048475 | 0.192848 |
| *Pseudomonas_E sp900187635* | M1 | -0.27235 | 0.06051 | 0.215148 |

Table S2. Results of standard univariate Cox regression and times selected in 100×10 cross validation of elastic net Cox model

| Characteratics | Times selected | Count/Event | HR | L95CI | H95CI | pvalue |
| --- | --- | --- | --- | --- | --- | --- |
| Age | 99 | (16/33) | 2.101629 | 1.055099 | 4.186188 | 0.034642 |
| *Ralstonia_pickettii_B* | 60 | (29/33) | 2.787375 | 0.975596 | 7.963809 | 0.055641 |
| *Limnohabitans_A_sp005789685* | 43 | (7/33) | 0.751863 | 0.308278 | 1.833727 | 0.530673 |
| *Bacillus_A_bombysepticus* | 39 | (5/33) | 1.059556 | 0.406605 | 2.761058 | 0.905763 |
| BVI | 39 | (14/33) | 2.09871 | 1.033714 | 4.260928 | 0.040195 |
| *Cutibacterium_granulosum* | 36 | (1/33) | 0.340351 | 0.046244 | 2.504963 | 0.289922 |
| Family_history | 33 | (3/33) | 0.362604 | 0.110217 | 1.192929 | 0.094995 |
| Location | 33 | (13/33) | 0.600849 | 0.29702 | 1.215474 | 0.156447 |
| *Sphingobium_yanoikuyae* | 33 | (1/33) | 0.304981 | 0.041554 | 2.238362 | 0.242938 |
| PNI | 24 | (23/33) | 1.430616 | 0.676077 | 3.027265 | 0.349074 |
| *QWOQ01_sp003669585* | 23 | (12/33) | 1.023312 | 0.499243 | 2.09751 | 0.949822 |
| *Sphingomonas_olei* | 19 | (5/33) | 0.851031 | 0.326619 | 2.21743 | 0.741298 |
| *Acinetobacter_guillouiae* | 16 | (7/33) | 0.650371 | 0.28149 | 1.502657 | 0.313997 |
| *Alcaligenes_phenolicus* | 11 | (4/33) | 0.94626 | 0.330256 | 2.711257 | 0.918083 |
| *Brevundimonas_sp002434505* | 11 | (7/33) | 1.10458 | 0.475816 | 2.564224 | 0.816943 |
| *Ralstonia_sp000620465* | 11 | (2/33) | 0.434541 | 0.103542 | 1.823664 | 0.254738 |
| *Brevundimonas_diminuta_A* | 9 | (4/33) | 0.75289 | 0.263667 | 2.149849 | 0.59597 |
| M_stage | 9 | (1/33) | 2.574108 | 0.334186 | 19.82735 | 0.364029 |
| CA19_9_upregulate | 8 | (25/33) | 0.881208 | 0.396793 | 1.957008 | 0.756067 |
| *Cutibacterium_acnes* | 8 | (2/33) | 0.401528 | 0.095459 | 1.688942 | 0.213162 |
| Gender | 8 | (18/33) | 0.820228 | 0.411994 | 1.632969 | 0.572698 |
| *Klebsiella_pneumoniae* | 8 | (20/33) | 0.699068 | 0.34464 | 1.417989 | 0.321134 |
| *Pelomonas_sp003963075* | 8 | (4/33) | 1.363964 | 0.468792 | 3.968488 | 0.568927 |
| *Pseudomonas_E_lundensis* | 8 | (21/33) | 1.190236 | 0.568985 | 2.489807 | 0.64374 |
| *Pseudomonas_E_sp900187635* | 8 | (31/33) | 3.120053 | 0.739271 | 13.16801 | 0.121436 |
| *Ralstonia_pickettii* | 8 | (19/33) | 1.084559 | 0.540705 | 2.175434 | 0.819204 |
| *Sphingomonas_aquatilis* | 8 | (22/33) | 0.70256 | 0.334561 | 1.475338 | 0.351021 |
| *Staphylococcus_aureus* | 8 | (16/33) | 1.121026 | 0.558978 | 2.248208 | 0.74763 |
| *BACL27_sp014190055* | 7 | (10/33) | 1.370074 | 0.642819 | 2.920111 | 0.414795 |
| *Dietzia_maris* | 7 | (1/33) | 0.476112 | 0.064002 | 3.541815 | 0.468572 |
| *Lawsonella_clevelandensis_A* | 7 | (5/33) | 0.939372 | 0.360511 | 2.447694 | 0.89815 |
| Other_malignancy | 7 | (3/33) | 0.91906 | 0.279116 | 3.026235 | 0.889596 |
| T_stage | 31 |  | 1.215211 | 0.817493 | 1.806422 | 0.335207 |
| Differentiation | 39 |  | 0.563336 | 0.305684 | 1.038155 | 0.065781 |
| Smoking | 8 |  | 1.01458 | 0.69237 | 1.486739 | 0.940815 |
| Stage | 8 |  | 1.199235 | 0.764242 | 1.881816 | 0.429326 |
| Hyperlipemia | 8 |  | 1.186989 | 0.807215 | 1.745438 | 0.383566 |
| Diabetes | 8 |  | 0.86256 | 0.553405 | 1.344422 | 0.5138 |
| N_stage | 9 |  | 1.198519 | 0.7384 | 1.945354 | 0.463696 |
| Alcohol | 8 |  | 0.959955 | 0.666643 | 1.38232 | 0.826124 |
| BMI_level | 8 |  | 0.966724 | 0.60629 | 1.541433 | 0.886947 |
| *Brevundimonas_diminuta* |  | (0/33) |  |  |  |  |
| *Citrobacter_braakii* |  | (0/33) |  |  |  |  |
| Antibiotics |  | (0/33) |  |  |  |  |
| Neoadjuvant_therapy |  | (0/33) |  |  |  |  |
| *Pseudomonas_E_fulva* |  | (0/33) |  |  |  |  |

Table S3. Represent reaction formula of differential microbial derived metabolites and microbial KO genes.

| ID | Reaction | Formula | KO | Enzyme | Substrate | Product |
| --- | --- | --- | --- | --- | --- | --- |
| R01288 | O-Succinyl-L-homoserine + Hydrogen sulfide <=> L-Homocysteine + Succinate | C01118 + C00283 <=> C00155 + C00042 | K07094 | pcrB  [EC:2.5.1.-] | _ | C00042 |
| R00132 | Carbonate <=> CO_2_ + H_2_O | C01353 <=> C00011 + C00001 | K07566 | tsaC, rimN, SUA5  [EC:2.7.7.87] | C01353 | _ |
| R01470 | sn-Glycero-3-phosphoethanolamine + H_2_O <=> Ethanolamine + sn-Glycerol 3-phosphate | C01233 + C00001 <=> C00189 + C00093 | K01126 | glpQ, ugpQ  [EC:3.1.4.46] | C01233 | C00093 |
| R00908 | beta-Alanine + 2-Oxoglutarate <=> 3-Oxopropanoate + L-Glutamate | C00099 + C00026 <=> C00222 + C00025 | K00823 | puuE  [EC:2.6.1.19] | C00099,  C00026 | _ |

Data S1. (separate file)

An .xls file including:

1. Table of microbiome reads quality control;
2. Table of microbial species detected in environment samples;
3. Table of differential abundant metabolites;
4. Table of all microbial species detected;
5. Table of metadata;
6. Table of overall survival information;
7. Results of MaASLin2 on KO level.
8. Species include in survival analysis.
9. Significant correlation of species-KO genes-metabolites.
